# Supplementary material for: Metamorphism of Venus as driver of crustal thickness and recycling
Source: Nat Commun. 2025 Mar 25;16:2905. doi: 10.1038/s41467-025-58324-1 (PMC11937330; doi:10.1038/s41467-025-58324-1)
Supplement: Supplementary file 1 — Description of Additional Supplementary Files [file 41467_2025_58324_MOESM1_ESM.pdf]

Description of Additional Supplementary Files

File name: Supplementary Data 1
Description: Tabulated densities calculated by Perple_X for peridotite, basalt, and alkali basalt for thermal gradients of 5-25 °C/km (shown in Figure 2) and computed mineral modes for peridotite, basalt, and alkali basalt on thermal gradients of 5-25 °C/km (shown in Figure 4).
